# High Serum Aspartate Aminotransferase, Underweight, and Weight Loss in Older People: Results of the KITCHEN-4

**DOI:** 10.3390/healthcare8020069

**Published:** 2020-03-25

**Authors:** Michi Shibata, Kei Nakajima

**Affiliations:** 1 School of Nutrition and Dietetics, Faculty of Health and Social Services, Kanagawa University of Human Services, 1-10-1 Heisei-cho, Yokosuka, Kanagawa 238-8522, Japan; sibat.nu@marianna-u.ac.jp; 2 Department of Nutrition, St. Marianna University School of Medicine, 2-16-1 Sugao, Miyamae-ku, Kawasaki, Kanagawa 216-8511, Japan; 3 Graduate School of Health Innovation, Kanagawa University of Human Services, Research Gate Building Tonomachi 2-A, 3-25-10 Tonomachi, Kawasaki, Kanagawa 210-0821, Japan; 4 Department of Endocrinology and Diabetes, Saitama Medical Center, Saitama Medical University, 1981 Kamoda, Kawagoe, Saitama 350-8550, Japan

**Keywords:** body mass index, exercise, lifestyle, weight loss, underweight

## Abstract

*Background:* Reduced muscle mass is frequently observed in older people and can lead to being underweight and/or weight loss (WL), but prediction and screening systems utilizing hematological biochemical parameters are limited. High serum aspartatSe aminotransferase in conjunction with normal serum alanine aminotransferase (HASNAL) can reflect systemic muscle damage. HASNAL and the incidence of being underweight concomitant with WL (UWWL) were investigated in the present 6-year community-based cohort study. *Methods:* Clinical parameters, lifestyle, the incidence of being underweight, ≥5% WL, and UWWL were investigated in 238,536 Japanese people aged 40–68 years who had normal serum alanine aminotransferase. HASNAL was defined as serum aspartate aminotransferase ≥30 U/L and serum alanine aminotransferase <30 U/L. The subjects were divided into a younger group (<55 years) and an older group (≥55 years). *Results:* After 6 years, overall body weight had increased by 0.4% in the younger group and decreased by 0.4% in the older group. In logistic regression analysis, in the younger group ≥5% WL was significantly associated with baseline HASNAL compared to baseline low serum aspartate aminotransferase (<20 U/L). In the older group, baseline HASNAL was significantly associated with being underweight, ≥5% WL, and UWWL, even after adjustment for potential confounders, and UWWL was inversely associated with regular exercise and daily alcohol consumption—both of which modified the aforementioned associations. *Conclusions:* Older subjects with HASNAL were at an increased risk of UWWL, possibly via skeletal muscle damage, which may be affected by common lifestyles.

## 1. Introduction

Geriatric people are predisposed to weight loss (WL), and in such patients, WL can be accelerated by reduced skeletal muscle mass, which is a specific feature of sarcopenia [1,2,3]. Being underweight and WL, which are characteristic features of frailty [3,4], are risk factors for mortality in the elderly [4,5]. The etiologies of sarcopenia and frailty can substantially overlap geriatric conditions upstream of disabling cascades [5], with accompanying reduced skeletal muscle mass and function, and potential malnutrition as indicated by being underweight [3,4]. To date, the determination of sarcopenia has required measurement of skeletal muscle mass, which is commonly achieved via dual energy x-ray absorptiometry, computed tomography, or magnetic resonance imaging [1,3], in conjunction with specific physical features such as reduced walking speed and/or reduced handgrip strength [1,3,4]. Such parameters are usually time-consuming to quantify, however, and doing so requires substantial medical staff resources. There is currently no hematological biomarker for the prediction or screening of sarcopenia, being underweight, or WL available for use in routine clinical practice, potentially resulting in a high prevalence of overlooked and undiagnosed patients in the early stages of being underweight, undergoing progressive WL, and/or sarcopenia, despite the general aging of the human population worldwide.

In the past decade, several studies have shown that low blood alanine aminotransferase (ALT) was associated with low fitness, frailty, and sarcopenia [6,7,8], although the mechanisms underlying these observations remain unknown. Aspartate aminotransferase (AST) is normally present in multiple organs including the liver, myocardium, and skeletal muscle [9,10,11], and high serum AST can reflect systemic muscle damage. In previous cross-sectional studies in two different populations [12,13], high AST in conjunction with normal ALT (HASNAL) was observed in older underweight individuals, and it was not substantially altered after adjustment for potentially confounding factors such as sex, smoking, and physical activity [12]. In addition, a low level of serum creatinine, a surrogate marker of skeletal muscle mass [14,15], was observed in men and women with HASNAL [13]. Notably however, the causal relationships between HASNAL and being underweight and/or undergoing WL remain uncharacterized. These relationships and the potential involvement of ALT were investigated in the current community-based 6-year cohort study.

## 2. Methods

### 2.1. Study Design

The present investigation was a composite study using health checkup data acquired in Japan as part of the Kanagawa Investigation of the Total Checkup Data from the National Database (KITCHEN), which is a broader study aimed at elucidating factors associated with cardiometabolic diseases. The design of that study is described in detail elsewhere [16]. Since 2008, all people living in Japan aged 40 to 74 years are supposed to undergo a yearly itemized health checkup managed by the Ministry of Health, Labour and Welfare (MHLW) [17]. The current study was approved by the ethics committee of Kanagawa University of Human Services (ID number 10-43) and the MHLW in Japan (ID number 121). To negate the identification of specific individuals, patient ages were categorized as 40–44, 45–49, 50–54, 55–59, 60–64, 65–69, or 70–74 years before the datasets were collated.

### 2.2. Subjects

The present cohort study used data derived from 593,517 people who underwent health checks twice, 6 years apart, the first time between April 2008 and March 2009, and the second time between April 2014 and March 2015. Subjects with baseline serum AST ≥ 200 U/L, ALT ≥ 30 U/L, and/or GGT ≥ 200 IU/L, and those with a baseline body mass index (BMI) < 18.5 or ≥ 25.0 were excluded. Subjects with serum ALT ≥ 30 U/L after 6 years were not enrolled, in order to exclude patients with high serum AST due to hepatic disease. A total of 238,536 people (113,764 men and 124,772 women) aged 40–68 years and of normal weight with complete data (except for HbA1c and past history of cerebrovascular disease) were enrolled in the present study. To evaluate subject age as a single numeric value, age groups (40–44, 45–49, 50–54, 55–59, 60–64, and 65–69 years) were transformed into substituted ages (s-age) corresponding to the medians in each age group, which were 42, 47, 52, 57, 62, and 67 years. In the analysis of the effects of age, the subjects were divided into two age groups, “younger” (<55 years, *n* = 129,077) and “older” (≥55 years, *n* =109,459). Because it has been reported that in most developed countries peak body weight is observed between the ages of 50 and 59 years, and that body weight begins to decline thereafter [18,19,20], we chose the mid-50s as a cut-off age.

### 2.3. Measurement of Clinical Parameters

BMI and other measurements were conducted in the morning after an overnight fast. Clinical biochemical parameters including AST, ALT, and GGT were measured using internal and external standards, in accordance with the HMWL [16]. Being underweight concomitant with WL (UWWL) was defined as exhibiting a BMI < 18.5 concomitant with a reduction in body weight of ≥5% compared to baseline body weight (≥5% WL) [3,21,22,23]. HASNAL was defined as AST ≥ 30 U/L concomitant with ALT < 30 U/L based on provisional reference ranges derived from Japanese populations [24]. Baseline serum AST was classified into three ranges, <20 U/L, 20–29 U/L, and ≥30 U/L. Because the distribution of serum AST was highly skewed and the concentration of serum AST was expressed as an integral number, dividing subjects into strictly defined tertile or quartile serum AST groups was problematic.

### 2.4. Statistical Analysis

Data are expressed as the mean ± standard deviation or the median and interquartile range. Continuous variables were investigated via analysis of variance, and categorical variables were investigated via the Cochran-Armitage test. The Cochran-Armitage test was used to investigate trends in the prevalence of being underweight, ≥5% WL, and UWWL in patients with different serum AST levels. Differences in changes in body weight during the 6 years after baseline in the three AST groups were analyzed via the Kruskal-Wallis test. Logistic regression models were used to assess associations between HASNAL and being underweight, ≥5% WL, and UWWL after 6 years with and without adjustment for potentially confounding baseline factors, yielding adjusted relative risks (ARRs), and 95% confidence intervals (CIs). Because the incidences of being underweight and UWWL were small (<5% in total) [25], adjusted odds ratios were nearly equivalent to ARRs. Potentially confounding baseline factors included sex, smoker status (current vs. noncurrent), alcohol consumption (frequencies and amounts), regular exercise (≥30 min per session at least twice weekly; yes vs. no), regular physical activity (walking or any equivalent amount of physical activity ≥ 1 h/day; yes vs. no), pharmacotherapies (hypertension, diabetes, dyslipidemia; yes vs. no), medical history of cardiovascular and/or renal disease (yes vs. no), age, BMI, serum triglyceride, GGT, and ALT. Statistical analyses were performed using SAS Analytical Suit (SAS-EG 7.1) Version 9.4 software (SAS Institute, Cary, North Carolina, USA). *p* < 0.05 was considered statistically significant.

## 3. Results

Baseline characteristics of the younger subjects in the three baseline AST categories are shown in Table 1. With the exceptions of HbA1c and a medical history of cerebrovascular disease, continuous parameters and the prevalence of categorical variables were positively associated with higher serum AST, though the increases in body weight and BMI were small. The baseline characteristics of older subjects are shown in Table 2. With the exceptions of BMI, body weight, and triglyceride, continuous parameters were positively associated with higher serum AST. With the exceptions of sex and pharmacotherapy for diabetes, the prevalence of categorical variables was negatively associated with higher serum AST.

Changes in body weight and the incidence of being underweight, ≥5% WL, and UWWL after 6 years are shown in Table 3. In younger subjects as a whole, there was a mean increase in body weight of 0.4 kg, but in the highest AST group (≥30 U/L) there was a decrease of 0.1 kg. Higher baseline AST was significantly associated with change in body weight and the incidence of ≥5% WL, but it was not significantly associated with being underweight or UWWL. In contrast, in older subjects’ body weight decreased by 0.4 kg. Change in body weight was significantly lower and the incidences of being underweight, ≥5% WL, and UWWL were significantly positively associated with higher baseline AST.

The results of logistic regression analysis of relationships between baseline serum AST and being underweight, ≥5% WL, and UWWL in the two age groups are shown in Table 4. In the younger group, low serum AST at the 6-year time-point (<20 U/L) and baseline serum AST ≥ 30 U/L (HASNAL) were significantly associated with the incidence of ≥5% WL after adjustment for all potential confounders. In the older group, low serum AST at the 6-year time-point, baseline serum AST 20–29 U/L, and baseline serum AST ≥ 30 U/L (HASNAL) were significantly associated with the incidences of being underweight, ≥5% WL, and UWWL (Model 1), after adjustment for all potential confounders. Notably, adjustment for regular exercise and drinking alcohol daily strengthened associations between AST and UWWL (Model 2a and Model 3) in the older group. When baseline serum AST ≥ 30 U/L was further divided into serum AST 30–34 U/L (*n* = 155) and ≥ 35 U/L (*n* = 59) in the older group, the ARR of baseline serum AST ≥ 35 U/L for UWWL was 1.51 ((1.21–2.02), *p* = 0.007) in Model 2 (data not shown). Serum ALT as a continuous variable was positively associated with the incidence of ≥5% WL in younger subjects (ARR 1.01, CI 1.00–1.01, *p* < 0.01), whereas in older subjects it was negatively associated with being underweight (ARR 0.99, CI 0.98–0.99) and UWWL (ARR 0.99, CI 0.98–1.00) (both *p* < 0.05), and positively associated with ≥ 5% WL (ARR 1.00, CI 1.00–1.01, *p* < 0.05). 

**Figure 1:**
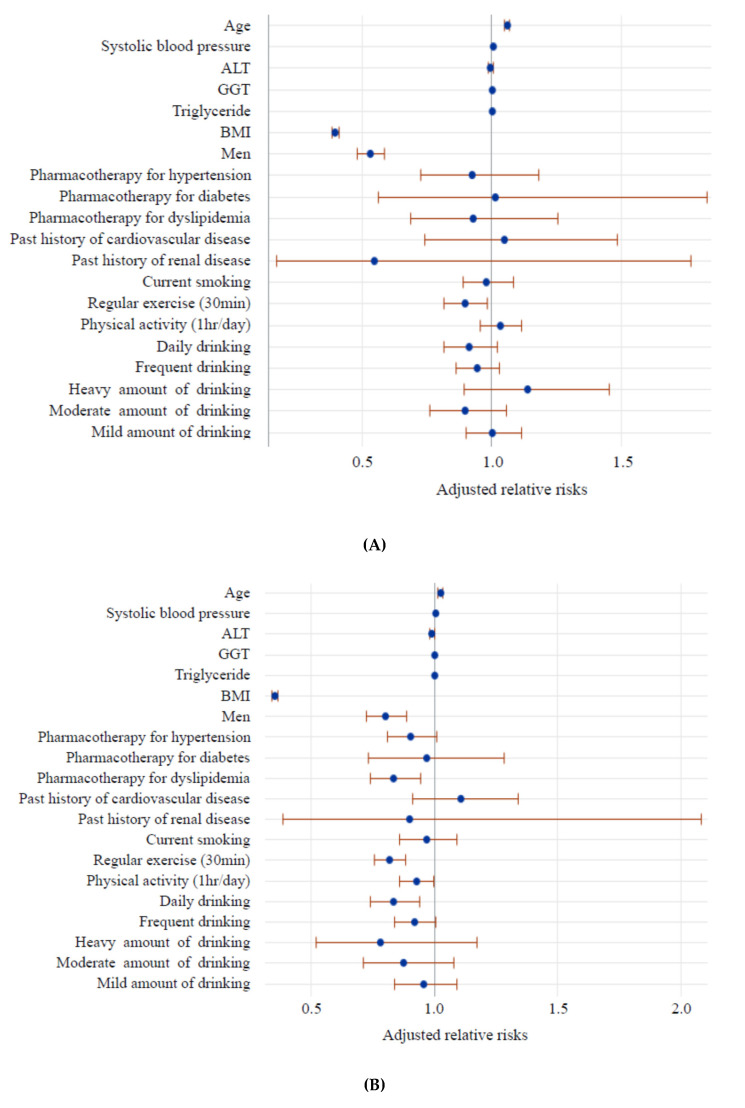
Adjusted relative risk of baseline factors for being underweight concomitant with weight loss after 6 years in younger (**A**) and older age groups (**B**). Each circle and horizontal bar represent Table 95. confidence interval after controlling for baseline confounders listed in the figure and baseline serum aspartate aminotransferase. Age, systolic blood pressure, alanine aminotransferase, GGT, triglyceride, and body mass index were treated as continuous variables. Dichotomized categories (pharmacotherapies for hypertension, diabetes, dyslipidemia, history of cardiovascular and/or renal disease, current smoking, regular exercise, physical activity) were evaluated vs. opposite corresponding conditions. Drinking every day and frequent drinking were evaluated vs. no drinking or hardly any drinking. Heavy (≥69 g ethanol), moderate (46–68 g ethanol), and mild (23–45 g ethanol) amounts of alcohol were evaluated vs. a lower amount of alcohol (<23 g ethanol).

The results of logistic regression analysis of baseline factors for UWWL are shown in Figure 1. In the younger group, BMI, being male, and habitual exercise were inversely associated with the incidence of UWWL. In the older group, BMI, being male, pharmacotherapy for dyslipidemia, regular exercise, and drinking alcohol daily were inversely associated with the incidence of UWWL. In both age groups, age was positively associated with UWWL.

## 4. Discussion

Generally, there is an increase in body weight with aging, and the peak prevalence of obesity is reportedly observed in individuals aged 50–59 years in most developed countries [18,19]. After 60 years of age, body weight and BMI begin to decline, as age-associated WL begins. Age-associated WL was likely to have begun in the younger group in the current study, which may have influenced the indistinguishable trends of changes in body weight with increasing AST (Table 3). In contrast, in the older subjects, body weights were markedly reduced in all three baseline serum AST-defined sub-groups, and the reductions were significantly associated with increasing baseline AST in all three subgroups (Table 3).

Indeed, aminotransferase abnormalities, particularly AST but not ALT, and elevated creatine kinase are common in the setting of rhabdomyolysis and post-exercise [26,27], which have been shown in relatively small studies of less than 300 subjects. In the present study consisting of approximately two hundred forty thousand subjects, older subjects with HASNAL were at an increased risk of being underweight, ≥5% WL, and UWWL. In younger subjects however—in whom age-associated WL had presumably begun—HASNAL was only significantly associated with ≥5% WL. Although it has not been confirmed that HASNAL truly reflects systemic skeletal muscle damage, the results of the current study suggest that skeletal muscle damage may be involved in underlying mechanisms pertaining to relationships between HASNAL and UWWL. Notably, AST was plenty enrolled in myocardium [9,11], and increased AST in circulation is observed in patients with cardiac infarction. Patients with acute myocardial infarction were unlikely to be enrolled in the present study however, because such patients are usually treated in emergency wards, although individuals with silent myocardial infarction without definite chest pain may have been included [28,29]. Alternatively, because it has been suggested that sarcopenia and heart failure share common pathophysiological pathways involving malnutrition, inflammation, oxidative stress, and humoral factors [30,31] a proportion of high serum AST may be attributable to chronic heart disease in older underweight people, which may be an associated feature of sarcopenia.

Being underweight or exhibiting WL after skeletal muscle damage indicate a reduction in muscle mass, and can be one of the main etiologies of sarcopenia or frailty [1,2,3,4,5], and the results of the current study may provide insight into the development of prediction and screening methods for the prevention and early detection of low body weight, WL, sarcopenia, and frailty. Therefore, with regard to prevention, older people with high AST without high ALT—i.e., HASNAL—may be prime targets for interventional strategies such as nutrition/exercise therapies and improvement of lifestyles, even though they may be of normal weight at the time of initial examination.

In most developed countries widespread incidences of being underweight, sarcopenia, and frailty reduce the welfare of older members of society due to increasing medical costs and nursing care [1,4]. Given that the determination of sarcopenia is usually time-consuming and requires considerable medical staff resources however, more concise and convenient screening methods, preferably utilizing commonly measured parameters, may be required in the rapidly aging societies of the future. To date, there is no hematological biochemical marker for being underweight, WL, or UWWL, which can reflect sarcopenia and/or frailty. In Japan and most developed countries worldwide, serum AST and ALT are routinely measured in the clinical setting and at annual regular checkups, and the process is inexpensive, time-efficient, and exhibits good reproducibility [16].

Although serum albumin is often reduced in patients with malnutrition, the use of hypoalbuminemia is limited in the clinical settings [32,33,34] because it is a result of malnutrition rather than a cause of it [32]. Circulating creatine kinase, aldolase, and myoglobin are indicators of skeletal muscle damage [35,36], but these parameters are generally only measured in the clinical setting if a patient complains of muscle pain or the physician suspects myositis. In serum creatinine, an indicator of muscle mass, the normal range is very narrow [15,37] which makes it difficult to distinguish abnormal values from normal values in the clinical settings.

It has been reported that lower ALT levels may reflect or predict sarcopenia, although the underlying mechanisms potentially involved remain unknown [6,7,8]. In the present study serum ALT was negatively associated with being underweight and UWWL in older subjects, whereas it was positively associated with ≥5% WL in both age groups. The former inverse association suggests that lower serum ALT is likely to be associated with UW and UWWL, which is consistent with the results of previous studies [6,7,8], but the latter positive association does not. Therefore, unlike HASNAL, low serum ALT did not predict WL irrespective of age group in the current study.

Intriguingly, the association between HASNAL and UWWL observed in the current study was modified by adjustment for regular exercise and drinking alcohol daily. In this study, regular exercise and regular physical activities, favorable lifestyle choices in terms of the prevention of sarcopenia [1,2,3], were inversely associated with the incidence of UWWL in older subjects. To an extent, this may be expected because regular exercise and frequent physical activity can maintain and increase the amount of skeletal muscle mass [1,3]. Conversely, the reason why daily alcohol consumption was inversely associated with UWWL is not known, and interestingly the association was not affected by the amount of alcohol consumed per day. A possible explanation is that alcohol may function as an appetizer, resulting in increased food intake and consequent weight maintenance or gain [38,39,40]. Alternatively, instead of the preventative effects of exercise, physical activity, and alcohol consumption, individuals who engage in such habits are more likely to avoid UWWL by way of other concomitant factors such as diet, education, and social status. Investigation of such factors was beyond the scope of the present study. It is also unclear whether pharmacotherapies for dyslipidemia, in which 3-hydroxy-3-methyl-glutaryl-CoA reductase inhibitor (statin) is frequently used, were inversely associated with UWWL in the older age group. Statins have been shown to possess several anti-inflammatory activities resulting in the beneficial reduction of atherosclerotic processes [41,42], which might also protect the inflammatory response in the skeletal muscle. On the other hand, because statin-associated muscle symptoms, including myopathy, are well-known adverse effects of the drug [43,44], the possibility warrants further study.

The present study had some limitations. It is unknown whether the causes of WL in the patients in the study were unintentional or intentional, and this may be of relevance with regard to the outcomes identified in the different age groups 6 years after the initial examination [21,22,23]. A proportion of the older subjects were likely to have engaged in a controlled diet and/or exercise for example, to prevent excess weight and metabolic syndrome. Dietary information was not available in the present study. Strictly speaking, unintentional WL should be distinguished from intentional WL. Because unintentional WL is generally much more prevalent in older people than in younger people however [23], the proportion of unintentional WL may have been larger in the older age group in the current study, and this warrants further investigation. Another potential limitation of the study is that the current criteria for HASNAL may not be applicable in certain populations because of differences in body weight, lifespan, and methods used to measure serum AST and ALT.

## 5. Conclusions

In the current study, older subjects with HASNAL were at an increased risk of being underweight, ≥5% WL, and UWWL, which are characteristic features of sarcopenia. These conditions may have involved skeletal muscle damage, which can be modified by lifestyle choices.

## Figures and Tables

**Table 1:** Baseline characteristics of younger subjects.

Serum AST Level:	≤19 U/L	20–29 U/L	≥30 U/L
N (%)	74,728 (57.9)	51,485 (39.9)	2864 (2.2)
Male, *n* (%)	35,227 (47.1)	32,208 (62.6)	1981 (69.2)
Substitutional age (years)	46.1 ± 4.0	47.0 ± 4.1	47.5 ± 4.1
Body mass index (kg/m²)	21.6 ± 1.7	21.8 ± 1.7	21.6 ± 1.7
Body weight (kg)	58.5 ± 8.3	60.1 ± 8.4	60.1 ± 8.1
Aspartate aminotransferase (U/L)	16.4 ± 2.0	22.6 ± 2.4	33.9 ± 6.8
Alanine aminotransferase (U/L)	14.2 ± 4.2	19.5 ± 4.7	23.0 ± 4.5
γ-glutamyl transferase (U/L)	22.7 ± 15.6	33.3 ± 24.7	47.7 ± 37.9
Triglyceride (mg/dL)	75 (55–106)	80 (58–117)	80 (55–122)
High-density lipoprotein cholesterol (mg/dL)	66.3 ± 15.6	68.8 ± 17.0	73.7 ± 18.9
Systolic blood pressure (mmHg)	115.3 ± 14.6	118.4 ± 15.0	121.3 ± 15.6
Diastolic blood pressure (mmHg)	71.7 ± 10.6	74.3 ± 10.9	76.4 ± 11.3
HbA1c (%)	5.4 ± 0.4	5.3 ± 0.4	5.3 ± 0.4
Available *n*	60,408	41,305	2251
Pharmacotherapy for			
Hypertension, *n* (%)	2441 (3.3)	2382 (4.6)	199 (7.0)
Diabetes, *n* (%)	532 (0.7)	304 (0.6)	26 (0.9)
Dyslipidemia, *n* (%)	1127 (1.5)	1338 (2.6)	84 (2.9)
Medical history			
Cardiovascular disease, *n* (%)	848 (1.1)	727 (1.4)	46 (1.6)
Cerebrovascular disease, *n* (%)	348 (0.5)	263 (0.5)	10 (0.4)
Available *n*	74,698	51,467	2862
Current smoker, *n* (%)	20,513 (27.5)	13,612 (26.4)	893 (31.2)
Alcohol intake			
Daily, *n* (%)	18,199 (24.4)	19,280 (37.5)	1508 (52.7)
High quantity (≥ 69 g ethanol/day), *n* (%)	2243 (3.0)	2549 (5.0)	258 (9.0)
Regular exercise (≥ 30 min per sessionat least twice/week), *n* (%)	14,538 (19.5)	13,239 (25.7)	1028 (35.9)
Physical activity (≥ 1 hour/day), *n* (%)	27,485 (36.8)	20,474 (39.8)	1302 (45.5)

*Note*. AST, aspartate aminotransferase. Data are presented as mean ± standard deviation, median and interquartile range, or *n* and percentage. All differences in continuous and categorical variables between the three groups were significant except for medical history of cerebrovascular disease (all *p* values < 0.001, analysis of variance and Cochran-Armitage test).

**Table 2:** Baseline characteristics of older subjects.

Serum AST Level:	≤19 U/L	20–29 U/L	≥30 U/L
N (%)	39,319 (35.9)	64,892 (59.3)	5248 (4.8)
Male, *n* (%)	17,295 (44.0)	24,745 (38.1)	2308 (44.0)
Age (years)	61.7 ± 4.1	62.4 ± 4.0	62.9 ± 4.0
Body mass index (kg/m²)	21.9 ± 1.7 **	21.8 ± 1.7 **	21.6 ± 1.7
Body weight (kg)	56.4 ± 7.9 **	55.2 ± 7.8 **	55.0 ± 7.7 *
Aspartate aminotransferase (U/L)	17.1 ± 1.8	23.1 ± 2.5	33.6 ± 6.4
Alanine aminotransferase (U/L)	14.7 ± 3.8	18.8 ± 4.4	22.8 ± 4.3
γ-glutamyl transferase (U/L)	24.6 ± 16.0	29.5 ± 21.6	39.4 ± 31.0
Triglyceride (mg/dL)	93 (69–127)	89 (66–123)	85 (62–121)
High-density lipoprotein cholesterol (mg/dL)	64.6 ± 15.8	68.6 ± 16.6	72.8 ± 18.3
Systolic blood pressure (mmHg)	125.3 ± 16.6	126.5 ± 16.7	127.9 ± 16.9
Diastolic blood pressure (mmHg)	76.3 ± 10.6	76.6 ± 10.5	77.1 ± 10.6
HbA1c (%)	5.6 ± 0.5	5.5 ± 0.4	5.5 ± 0.4
Available *n*	34,531	57,707	4713
Pharmacotherapy for			
Hypertension, *n* (%)	7535 (19.2)	12,432 (19.2)	1079 (20.6)
Diabetes, *n* (%)	1273 (3.2)	1351 (2.1)	106 (2.0)
Dyslipidemia, *n* (%)	3960 (10.1)	8825 (13.6)	768 (14.6)
Medical history			
Cardiovascular disease, *n* (%)	1515 (3.9)	2642 (4.1)	246 (4.7)
Cerebrovascular disease, *n* (%)	801 (2.0)	1242 (1.9)	100 (1.9)
Available *n*	39,282	64,848	5246
Current smoker, *n* (%)	7859 (20.0)	8514 (13.1)	729 (13.9)
Alcohol intake			
Daily, *n* (%)	9505 (24.2)	17,708 (27.3)	2011 (38.3)
High quantity (≥69 g ethanol/day), *n* (%)	540 (1.4)	1036 (1.6)	172 (3.3)
Regular exercise (≥30 min per sessionat least twice/week), *n* (%)	14,245 (36.2)	27,128 (41.8)	2544 (48.5)
Physical activity (≥1 h/day), *n* (%)	18,744 (47.7)	34,150 (52.6)	3030 (57.7)

*Note*. AST, aspartate aminotransferase. Data are presented as mean ± standard deviation, median and interquartile range, or *n* and percentage. All differences in continuous and categorical variables between the three groups were significant except for pharmacotherapy for hypertension and medical history of cerebrovascular disease (all *p* values < 0.0001, analysis of variance and Cochran-Armitage test). * *p* < 0.05, ** *p* < 0.0001 vs. younger subjects (*t*-test).

**Table 3:** Changes in body weight and the incidences of being underweight, ≥5% weight loss, and being underweight concomitant with weight loss.

Baseline Serum AST Category	Total	≤19 U/L	20–29 U/L	≥30 U/L
Younger group				
Change in body weight (kg)	0.4 (−1.6 to 2.4)	0.6 (−1.4 to 2.6)	0.2 (−1.8 to 2.1)	−0.1 (−2.0 to 1.9)
UW, *n* (%)	4733 (3.7)	2758 (3.7)	1844 (3.6)	131 (4.6)
≥5% WL, *n* (%)	18,017 (14.0)	9757 (13.1)	7805 (15.2)	455 (15.9)
UWWL, *n* (%)	3166 (2.5)	1841 (2.5)	1243 (2.4)	82 (2.9)
				
Older group				
Change in body weight (kg)	−0.4 (−2.2 to 1.4)	−0.3 (−2.2 to 1.5)	−0.5 (−2.2 to 1.3)	−0.6 (−2.3 to 1.1)
UW, *n* (%)	4593 (4.2)	1423 (3.6)	2882 (4.4)	288 (5.5)
≥ 5% WL, *n* (%)	21,521 (19.7)	7423 (18.9)	13,001 (20.0)	1097 (20.9)
UWWL, *n* (%)	3459 (3.2)	1086 (2.8)	2159 (3.3)	214 (4.1)

*Note*. AST, aspartate aminotransferase; UW, underweight; WL, weight loss; UWWL, underweight concomitant with weight loss. Data are presented a median and interquartile range or *n* and percentage. All differences in changes in body weight and categorical variables between three groups were significant, except UW and UWWL in the younger group (all *p* < 0.0001, Kruskal-Wallis test and Cochran-Armitage test).

**Table 4:** Relative risk and 95% confidence intervals of associations between baseline serum aspartate aminotransferase and being underweight, ≥5% weight loss, and being underweight concomitant with weight loss.

Baseline Serum AST Category	≤19 U/L	20–29 U/L	≥30 U/L
Younger group			
UW			
Model 1	1 (ref)	0.97 (0.91–1.03)	1.25 (1.05–1.50) *
Model 2	1 (ref)	1.07 (0.98–1.15)	1.20 (0.97–1.48)
≥5% WL			
Model 1	1 (ref)	1.19 (1.15–1.23) ***	1.26 (1.14–1.39) ***
Model 2	1 (ref)	1.11 (1.07–1.15) ***	1.14 (1.02–1.27) *
UWWL			
Model 1	1 (ref)	0.98 (0.91–1.05)	1.17 (0.93–1.46)
Model 2^a^	1 (ref)	1.07 (0.98–1.17)	1.14 (0.88–1.46)
Model 2	1 (ref)	1.08 (0.99–1.18)	1.16 (0.90–1.49)
Older group			
UW			
Model 1	1 (ref)	1.24 (1.16–1.32) ***	1.55 (1.36–1.76) ***
Model 2	1 (ref)	1.10 (1.02–1.19) *	1.28 (1.09–1.50) **
≥5% WL			
Model 1	1 (ref)	1.08 (1.04–1.11) ***	1.14 (1.06–1.22) **
Model 2	1 (ref)	1.06 (1.02–1.10) **	1.14 (1.05–1.23) **
UWWL			
Model 1	1 (ref)	1.21 (1.13–1.31) ***	1.50 (1.29–1.74) ***
Model 2^a^	1 (ref)	1.07 (0.98–1.17)	1.21 (1.02–1.44) *
Model 2	1 (ref)	1.10 (1.01–1.20) *	1.27 (1.07–1.52) **

*Note*. AST, aspartate aminotransferase; UW, underweight; WL, weight loss; UWWL, underweight concomitant with weight loss. Model 1: Unadjusted. Model 2: Adjusted for age, sex, use of anti-hypertensive therapy, history of cardiovascular disease, daily alcohol consumption, and regular exercise (≥ 30 min exercise per session > 2 times per week vs. less frequent exercise), body mass index, systolic blood pressure, triglyceride, serum aspartate aminotransferase, serum GGT, and smoking status. All adjustments for potential confounding factors were made using the baseline values. Model 2^a^: Model 2 excluding adjustment for daily alcohol consumption, regular exercise, and physical activity. * *p* < 0.05, ** *p* < 0.01, *** *p* < 0.0001.
